# Supplementary material for: Population analysis of Legionella pneumophila reveals a basis for resistance to complement-mediated killing
Source: Nat Commun. 2021 Dec 9;12:7165. doi: 10.1038/s41467-021-27478-z (PMC8660822; doi:10.1038/s41467-021-27478-z)
Supplement: Supplementary file 1 — Supplementary Information [file 41467_2021_27478_MOESM1_ESM.pdf]

Wee et al, Population analysis of *Legionella pneumophila* reveals a basis for resistance to complement-mediated killing

Supplementary Information

Supplementary Fig. 1

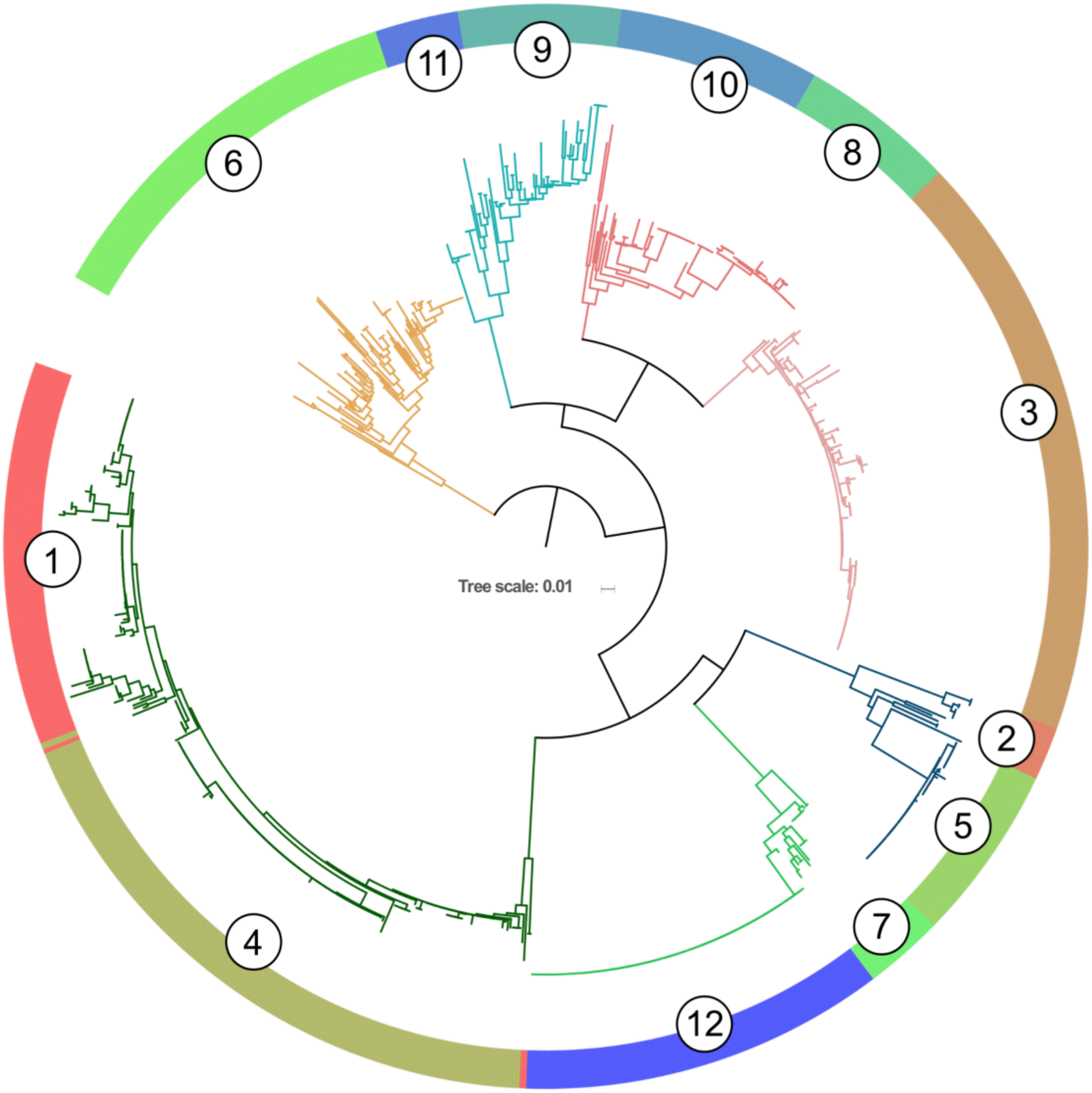

Supplementary Fig. 1. Maximum likelihood phylogenetic tree with BAPS clusters indicated. Each of the 7 major Maximum-Likelihood phylogenetic clades (branches coloured corresponding to Figure 1)

that define the *L. pneumophila* subsp. *pneumophila* population structure is supported by at least 1 and a maximum of 2 Bayesian Analysis of Population Structure (BAPS) clusters determined from the core SNP alignment. The tree scale indicates the number of substitutions per site.

Supplementary Fig. 2

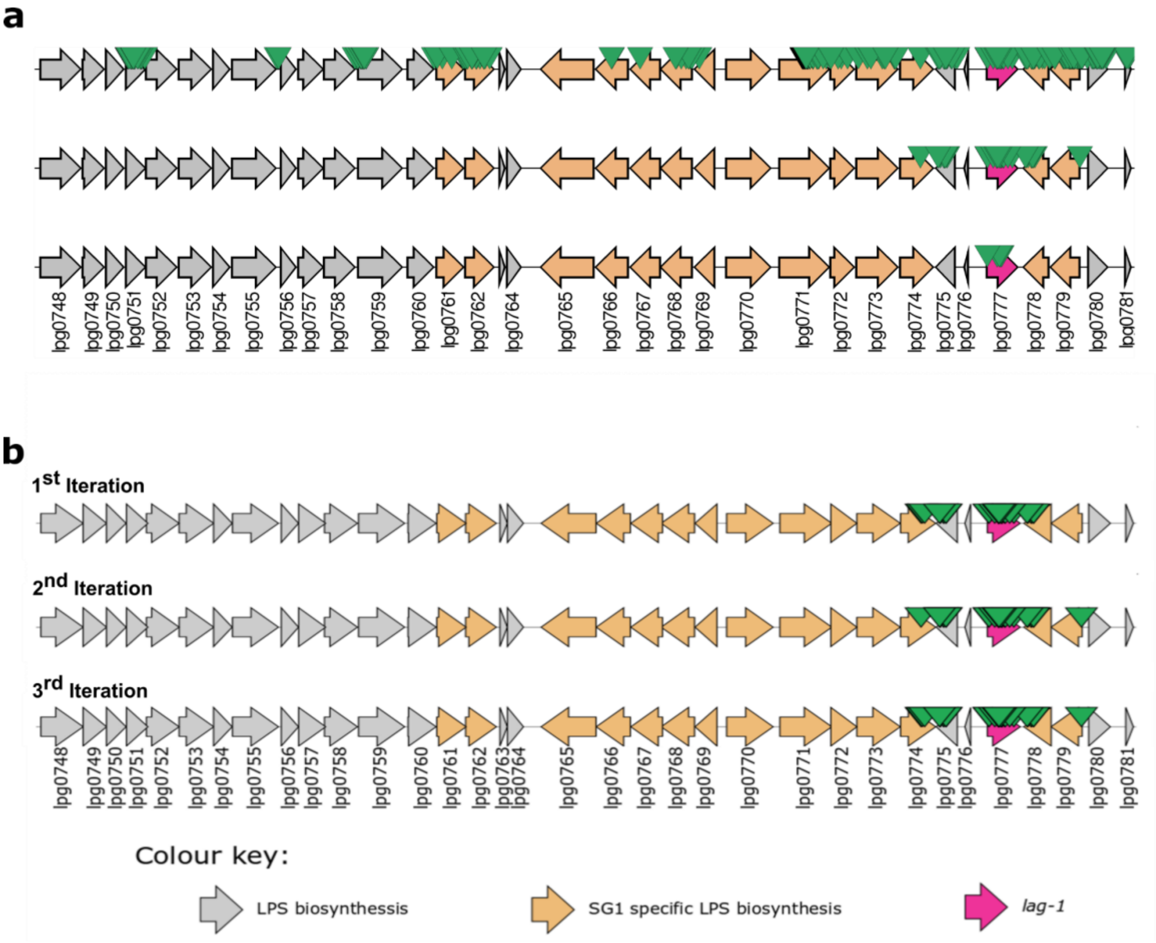

Supplementary Fig. 2. Localisation of significantly over-represented *k*-mers (indicated by green arrows) detected with SEER to the lipopolysaccharide (LPS) gene cluster (*lpg0748-lpg0781*) relative to the Philadelphia 1 reference genome. a) From top to bottom, the effect of subsampling using increasingly stringent minimum phylogenetic distance thresholds (Top: 0.0001 [≈180 SNPs], Middle: 0.001 [≈1800] and Bottom: 0.01 [≈18000 SNPs]). b) The 0.001 minimum distance subsampling was performed 3 times independently to demonstrate that the distribution of k-mers was consistent across iterations. Genes associated with SG1 specific LPS biosynthesis are represented in yellow, *lag-1* in pink and other genes involved in the LPS biosynthesis in grey.

Supplementary Fig. 3

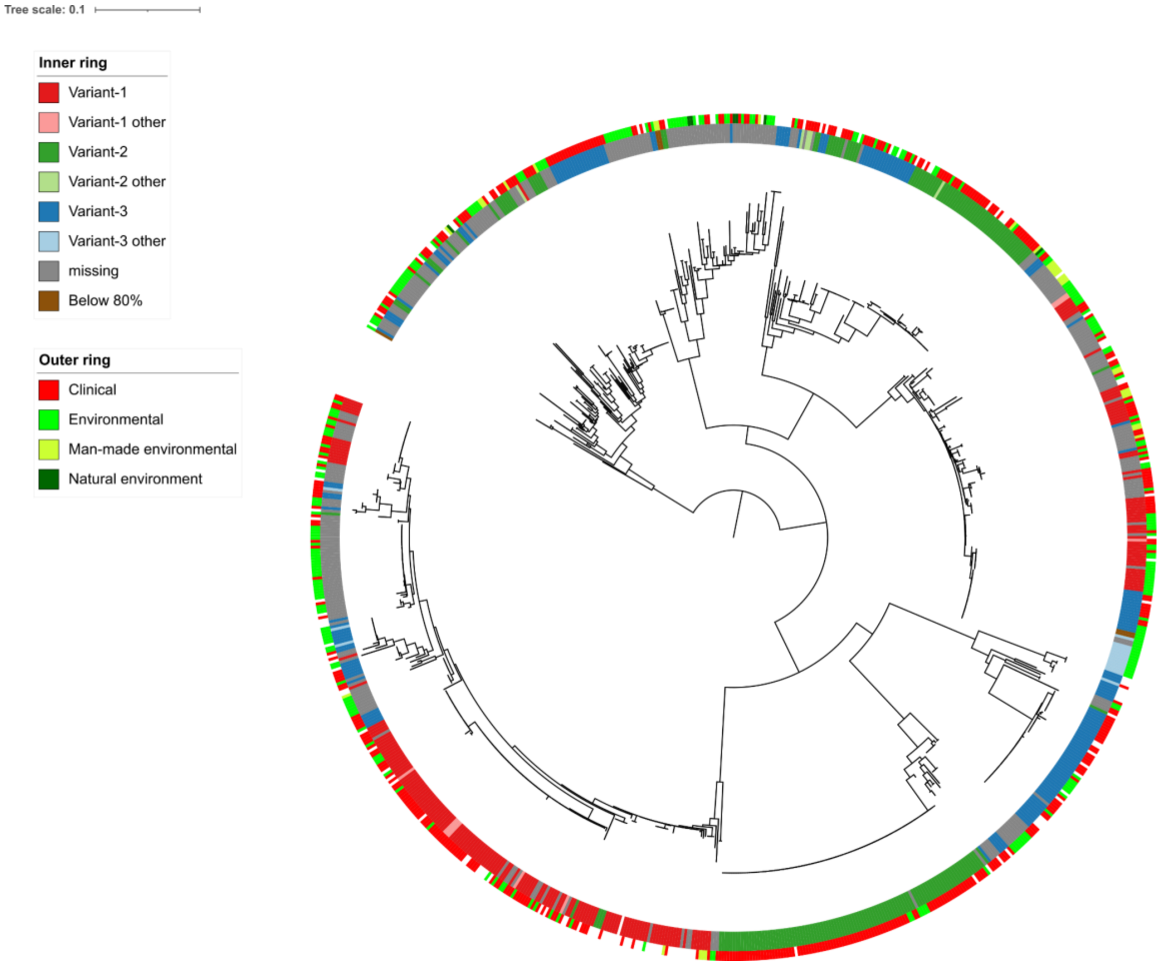

Supplementary Fig. 3. Core-genome SNP-based phylogenetic tree indicating the distribution of the 3 major allelic variants of *lag-1* across Sg-1 strains (inner ring) and the isolation origin of the strains (outer ring). Inner ring: Variant 1 (Philadelphia, Red), Variant 2 (Arizona, Green) and Variant 3 (Corby, Blue). Minor *lag-1* alleles that are within >99% nucleotide sequence identity to a major variant are indicated with lighter shades. Isolates not containing *lag-1* are colored grey. Outer ring: clinical strains (red), environmental strains (green), with strains isolated from man-made environments in light green and from natural environments in darker green. Strains of unknown isolation origin are indicated in white. The tree scale indicates the number of substitutions per site.

Supplementary Fig. 4

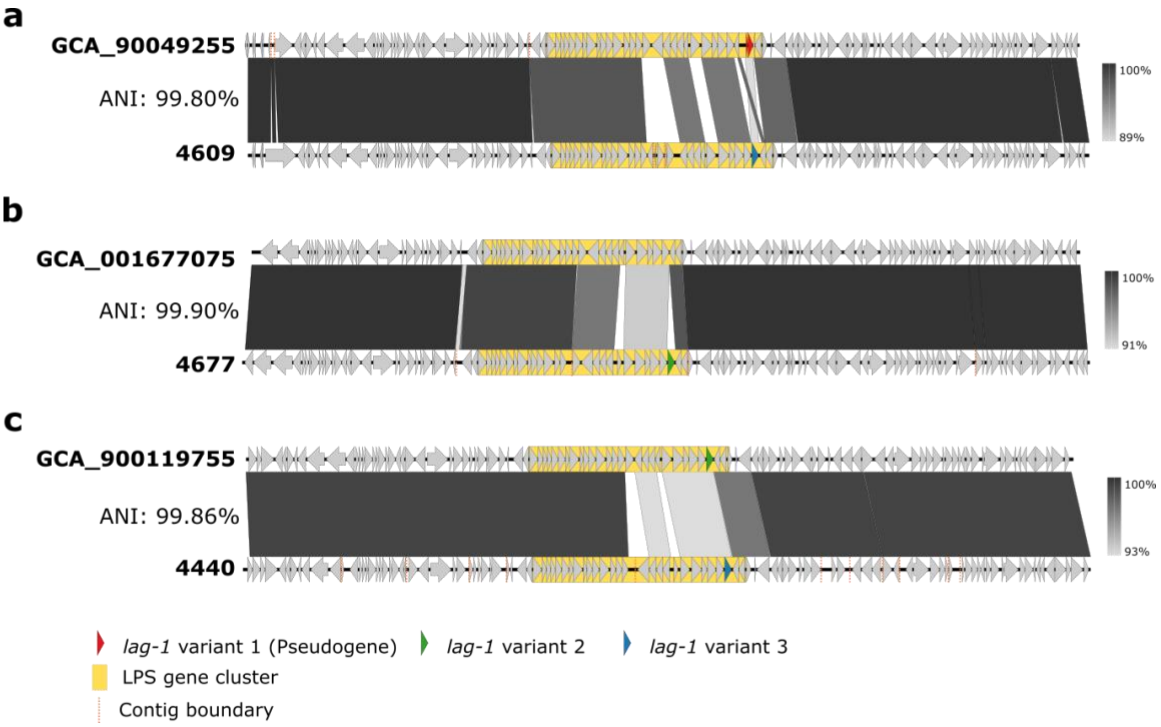

Supplementary Fig. 4. Recombination events driving replacement of *lag-1* variants. Pairwise alignment of genomic region flanking the LPS gene cluster indicating homologous recombination events between closely related genomes associated with switching between a disrupted a) *lag-1* variant 1 and a *lag-1* variant 3 , a b) *lag-1* LPS cluster with *lag-1* variant 2 , and a c) *lag-1* variant 2 with a *lag-1* variant 3. The average nucleotide identity (ANI) between genomes is shown on the left of the alignment and the level of nucleotide identity of the region shown is indicated by the grey scale on the right of each alignment.

Supplementary Fig. 5

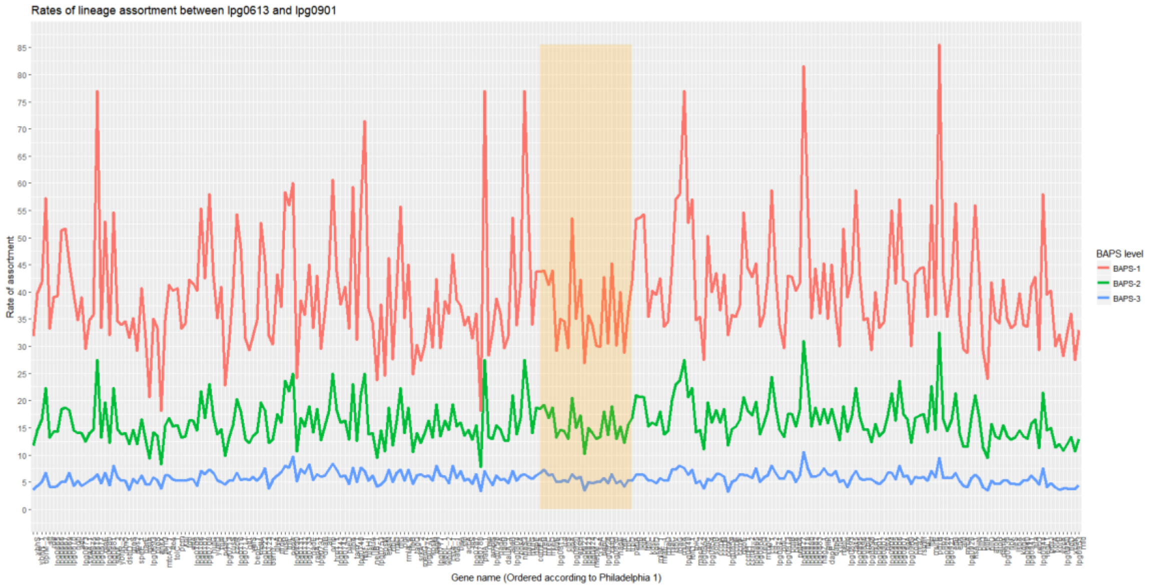

Supplementary Fig. 5. Rates of assortment across LPS biosynthesis clusters (highlighted) relative to surrounding genes. The rate of assortment is calculated by comparing the number of unique combinations between alleles, as defined by fastGEAR, and the BAPS populations clusters, normalised by the number of possible alleles for each gene.

Supplementary Fig. 6

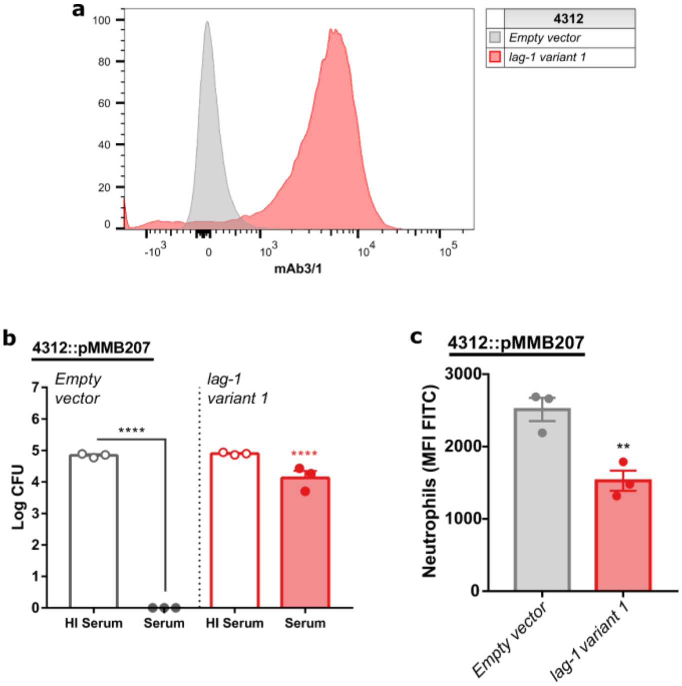

Supplementary Fig. 6. *lag-1* expression confers serum complement resistance and decreases human neutrophil phagocytosis to *L. pneumophila* strain 4312 with non-functional *lag-1* gene. a) Detection of mAb 3/1 epitope by flow cytometry in isogenic *L. pneumophila* expressing the *lag-1* variant 1 or containing the empty vector. b). Isogenic strains were incubated with human serum or heat inactivated serum for 1 h at 37°C. Each point represents an average of triplicate CFU counts of a single sera donor (n=3). Bars represent mean+SEM. One-way ANOVA, Tukey's multiple comparisons test ****p<0.0001 c) *L. pneumophila* isolates were stained with FITC and pre-incubated with 10% human serum 15 min prior incubation with human neutrophils for 30 min. Phagocytosis was evaluated by measuring neutrophils FITC fluorescence by flow cytometry. Each point respresents a technical replicate for a single donor. Data representative of three independent experiments. Two-tailed *t*-test **p=0.0098

Supplementary Fig. 7

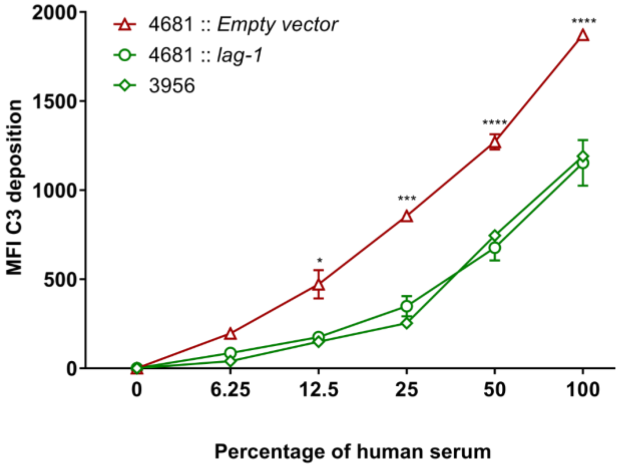

Supplementary Fig. 7. Deposition of human C3 on the surface of *L. pneumophila* is dependent on *lag-1* expression. Fixed bacteria were incubated with serial dilutions of normal human serum for 2 hours prior incubation with FITC conjugated anti-C3 Fab antibodies. Strains expressing *lag-1* in green and *lag-1* negative strain in red. Mean +/- SD, (n=2 serum donors). Two-way ANOVA multiple comparison test, *p=0.0138, ***p=0.0001, ****p<0.0001.

Supplementary Fig. 8

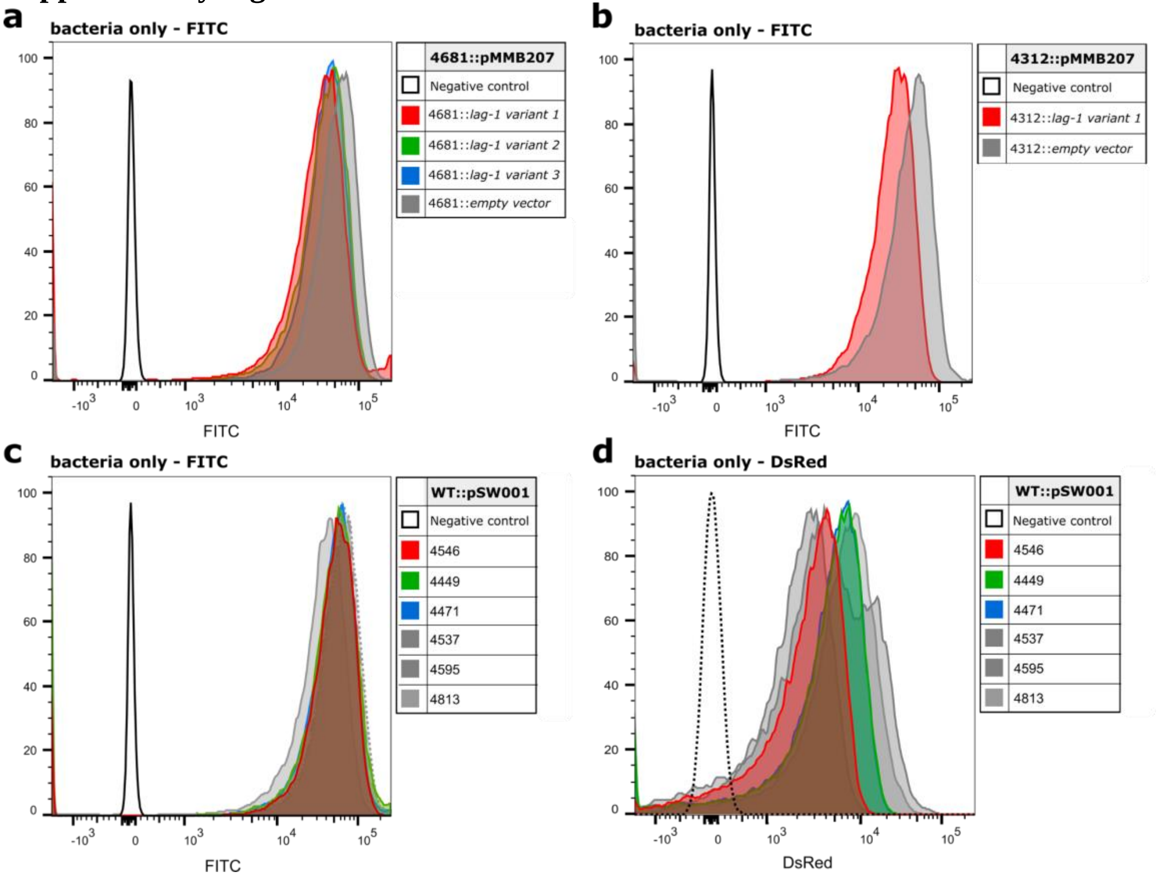

Supplementary Fig. 8. a to c) Fluorescent-labelled *L. pneumophila* strains exhibit similar levels of FITC MFI. FITC MFI of *L. pneumophila* isogenic strains (a and b) and WT strains transformed with a plasmid expressing DsRed (c) that express *lag-1* variant 1 (red), variant 2 (green), variant 3 (blue) or are negative for *lag-1* expression (grey). Non-FITC labelled bacteria were used as negative control. **d) *L. pneumophila* strains transformed with pSW001 plasmid express levels of fluorescent DsRed that are independent of *lag-1* expression.** DsRed MFI of WT *L. pneumophila* strains that express *lag-1* variant 1 (red), variant 2 (green), variant 3 (blue) or are negative for *lag-1* expression (grey).

Supplementary Fig. 9

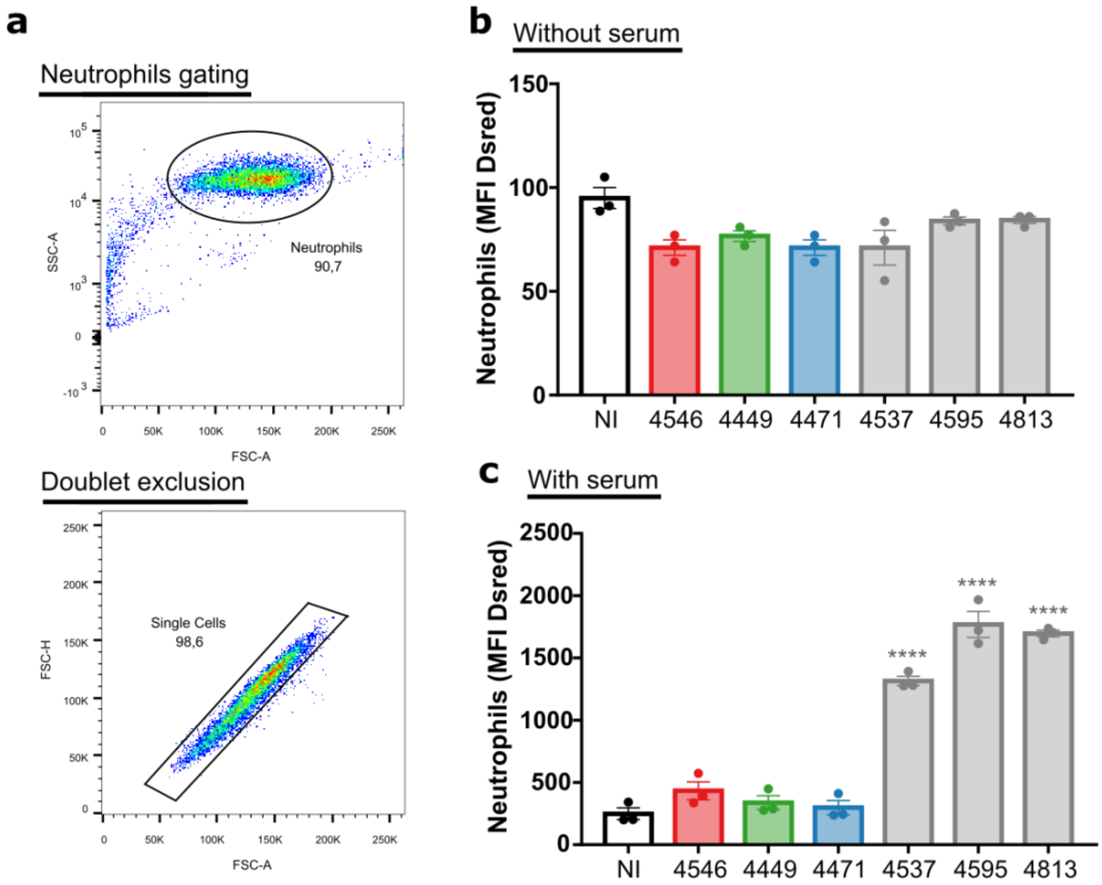

Supplementary Fig. 9. a) Representative pseudocolour plots and gating strategy for flow cytometry analysis of infected neutrophils. Neutrophil population was defined by FSC/SSC profile and doublets excluded using FSC-H/FSC-A ratios. Neutrophils MFI was quantified in the single cell population. **b and c) Neutrophil phagocytosis of *L. pneumophila* strains is serum dependent.** Phagocytosis was evaluated by measuring DsRed fluoresce of neutrophils infected with WT strains for *lag-1* gene transformed with pSW001 plasmid in the absence (b) or presence (c) of serum by flow cytometry. *L. pneumophila* strains expressing *lag-1* variant 1, 2 or 3 are represented in red, green or blue, respectively. Strains that do not express a functional *lag-1* gene are represented in gray. Non-infected (NI) neutrophils are represented in black. Bars represent mean+SEM. b) Each point represents and individual donor (n=3 biological replicates) b) Each point represents a technical replicate of the same donor (n=3 technical replicates). Graph representative of five independent experiments. One-way ANOVA, Dunnett's multiple comparisons test to non-infected control. ****p<0.0001

Supplementary Fig. 10

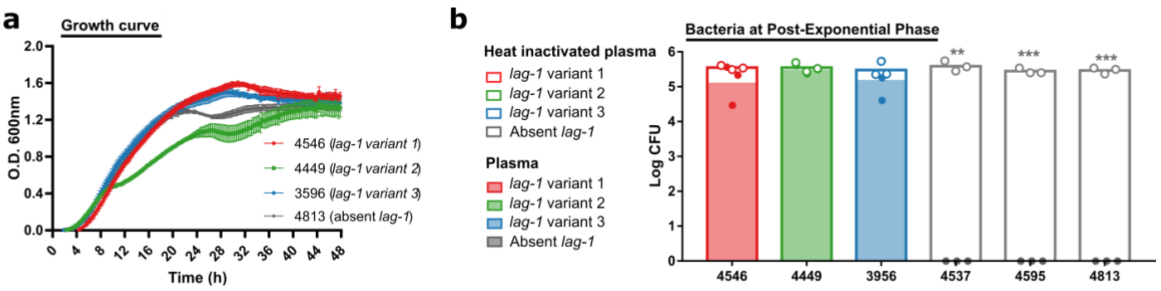

Supplementary Fig. 10. ***lag-1*** **expression confers serum complement resistance in** ***L. pneumophila*** **isolates at post-exponential phase.** a) Growth curves of *lag-1* positive and negative isolates, indicating each strain to be at post-exponential phase after 40 h of growth and an $O.D_{600} \approx 1.4$ approximately b) WT *L. pneumophila* isolates containing allelic variants 1, 2, and 3 of *lag-1* (depicted in red, green, and blue, respectively) or lag-1-negative (depicted in grey) cultured to post-exponential phase were incubated with human serum (coloured bars and dots) or heat inactivated serum (open bars and dots) for 1 h at 37°C. Each point represents an average of triplicate CFU counts of a single serum donor (n=3). One-way ANOVA, Sidak's multiple comparisons test **p=0.0014, ***p=0.0004 (for 4595) and ***p=0.0005 (for 4813).

Supplementary Table 1. Gene loci enriched among clinical isolates. a) Loci in the LPS biosynthesis cluster (*lpg0748-lpg0781* in Philadelphia-1) with mapped *k*-mers enriched among clinical isolates. **b)** Filtered hits of a pan-GWAS analysis using SCOARY showing genes that are significantly associated with clinical isolates. The following thresholds we used for filtering hits, as calculated by the SCOARY algorithm: Bonferroni p-value: <0.05 (Adjusted p-values using Bonferroni's method for multiple comparisons applied after performing a binomial test of the null hypothesis that the presence/absence of this gene is unrelated to clinical/environmental origin), Benjamini-Hochberg p-value: <0.05 (p-values adjusted using the Benjamini-Hochberg's correction for multiple comparisons) and Empirical p-value: <0.05 after 500 permutations (As described in the SCOARY documentation: "Empirical p-values are returned as (r + 1)/(N + 1) where N is the number of permutations (N=500), and r is the number of test statistics observed to be higher or equal to the unpermuted statistic").

a)

**Philadelphia-1 locus tag**	***k*-mers**
lpg0751	112
lpg0752	8
lpg0755	3
lpg0758	2
lpg0759	28
lpg0760	2
lpg0761	32
lpg0762	49
lpg0766	2
lpg0767	1
lpg0768	11
lpg0769	3
lpg0771	119
lpg0772	8
lpg0773	12
lpg0774	1
lpg0775	30
lpg0777	22
lpg0778	34
lpg0779	147
lpg0780	30
lpg0781	17

b)

**Philadelphia-1**	**Lens**	**SG-1 specific**	**Annotation**	**Sensitivity / Specificity**	**Odds ratio**	**Bonferroni p**	**Benjamini -Hochberg p**	**Empirical p**
lpg0777	*lpl0816*	yes	*O*-acetyltransferase, *lag-1*	69.4 / 74.3	6.56	9.74E-11	9.74E-11	2.00E-03
lpg0779	*lpl0818*	yes	putative glycosyltransferase (ORF 1)	88.1 / 41.2	5.2	5.13E-05	2.35E-05	3.79E-02
lpg0780	*lpl0819*	<90% present in non-SG-1	Beta-lactamase hydrolase-like protein (ORF 0)	87.5 / 41.9	5.05	7.04E-05	2.35E-05	2.59E-02
	lpl0815 (lpg0774)	yes	hypothetical protein (ORF3)	29.4 / 94.6	7.28	1.54E-04	3.84E-05	2.00E-03
lpg0767	*lpl0808*	yes	Stress response kinase A (ORF 10)	85.6 / 41.9	4.29	6.23E-04	6.92E-05	4.79E-02

Supplementary Table 2. A distance matrix indicating the percentage nucleotide identity divergence between representative allelic variants of *lag-1* sequences identified in the dataset.

Lag-1 variant group & name		Number of genomes	Full length?		4713	GCA_900049255	4424	3646-MajorVariant1	4312	4408	GCA_900073025	GCA_900060695	4317	4002-MajorVariant2	GCA_001600995	GCA_001600985	GCA_001601155	4369	GCA_001601175	4568	4376	4658	4016	3640-MajorVariant3	GCA_001601005	4418	4451	4365-MinorVariant1
Group 1	Philadelphia	1		4713																								
		2		GCA_900049255	0%																							
		1		4424	0%	0%																						
		199	Yes	3646-MajorVariant1	0%	0%	0%																					
		2		4312	0%	0%	0%	0%																				
		1		4408	0%	0%	0%	0%	0%																			
		1	Yes	GCA_900073025	0%	0%	0%	0%	0%	0%																		
		3	Yes	GCA_900060695	0%	0%	0%	0%	0%	0%	0%																	
Group 2	Arizona	1	Yes	4317	5%	5%	4%	5%	5%	5%	5%	5%																
		196	Yes	4002-MajorVariant2	5%	5%	4%	5%	5%	5%	5%	5%	0%															
Group 3b	*(On the branch leading to Group 3)*	1		GCA_001600995	8%	8%	9%	8%	8%	8%	8%	8%	9%	9%														
		1		GCA_001600985	9%	9%	9%	9%	9%	9%	9%	8%	10%	10%	0%													
		1		GCA_001601155	9%	9%	9%	9%	9%	9%	9%	9%	10%	10%	0%	0%												
Group 2b		3	Yes	4369	7%	7%	7%	7%	7%	7%	7%	7%	6%	6%	10%	10%	10%											
Group 3	Corby	1		GCA_001601175	9%	9%	9%	9%	9%	9%	9%	8%	10%	10%	0%	0%	0%	10%										
		2		4568	11%	10%	9%	10%	10%	10%	11%	10%	11%	11%	0%	0%	0%	11%	0%									
		1		4376	10%	10%	9%	10%	10%	10%	10%	10%	11%	11%	0%	0%	0%	11%	0%	0%								
		1		4658	10%	10%	9%	10%	10%	10%	10%	10%	11%	11%	0%	0%	0%	11%	0%	0%	0%							
		1		4016	10%	10%	9%	10%	10%	10%	10%	10%	10%	10%	0%	0%	0%	11%	0%	0%	0%	0%						
		179	Yes	3640-MajorVariant3	10%	10%	9%	10%	10%	10%	10%	10%	10%	10%	0%	0%	0%	11%	0%	0%	0%	0%	0%					
		1		GCA_001601005	10%	10%	9%	10%	10%	10%	10%	10%	10%	10%	0%	0%	0%	11%	0%	0%	0%	0%	0%	0%				
		1	Yes	4418	10%	10%	9%	10%	10%	10%	10%	10%	10%	10%	0%	0%	0%	11%	0%	0%	0%	0%	0%	0%	0%			
		1	Yes	4451	10%	10%	9%	10%	10%	10%	10%	10%	11%	11%	0%	0%	0%	11%	0%	0%	0%	0%	0%	0%	0%	0%		
		13		4365-MinorVariant1	10%	10%	9%	10%	10%	10%	10%	10%	11%	11%	0%	0%	0%	11%	0%	0%	0%	0%	0%	0%	0%	0%	0%	
