# Supplementary material for: Population analysis of Legionella pneumophila reveals a basis for resistance to complement-mediated killing
Source: Nat Commun. 2021 Dec 9;12:7165. doi: 10.1038/s41467-021-27478-z (PMC8660822; doi:10.1038/s41467-021-27478-z)
Supplement: Supplementary file 3 — Description of Additional Supplementary Files [file 41467_2021_27478_MOESM3_ESM.pdf]

Description of Additional Supplementary Files

File Name: Supplementary Data 1
Description: Strain list and metadata for genomes employed in the study

File Name: Supplementary Data 2
Description: Relative reassortment rates of genes conserved in at least 30% of genomes
