# Supplementary material for: Population analysis of Legionella pneumophila reveals a basis for resistance to complement-mediated killing
Source: Nat Commun. 2021 Dec 9;12:7165. doi: 10.1038/s41467-021-27478-z (PMC8660822; doi:10.1038/s41467-021-27478-z)
Supplement: Supplementary file 6 — Reporting Summary [file 41467_2021_27478_MOESM6_ESM.pdf]

nature research

Corresponding author(s): J. Ross Fitzgerald

Last updated by author(s): 10-11-2021

Reporting Summary

Nature Research wishes to improve the reproducibility of the work that we publish. This form provides structure for consistency and transparency in reporting. For further information on Nature Research policies, see our Editorial Policies and the Editorial Policy Checklist.

Statistics

For all statistical analyses, confirm that the following items are present in the figure legend, table legend, main text, or Methods section.

n/a	Confirmed	
☐	☒	The exact sample size (*n*) for each experimental group/condition, given as a discrete number and unit of measurement
☐	☒	A statement on whether measurements were taken from distinct samples or whether the same sample was measured repeatedly
☐	☒	The statistical test(s) used AND whether they are one- or two-sided<br>*Only common tests should be described solely by name; describe more complex techniques in the Methods section.*
☒	☐	A description of all covariates tested
☐	☒	A description of any assumptions or corrections, such as tests of normality and adjustment for multiple comparisons
☐	☒	A full description of the statistical parameters including central tendency (e.g. means) or other basic estimates (e.g. regression coefficient) AND variation (e.g. standard deviation) or associated estimates of uncertainty (e.g. confidence intervals)
☐	☒	For null hypothesis testing, the test statistic (e.g. *F*, *t*, *r*) with confidence intervals, effect sizes, degrees of freedom and *P* value noted<br>*Give P values as exact values whenever suitable.*
☒	☐	For Bayesian analysis, information on the choice of priors and Markov chain Monte Carlo settings
☒	☐	For hierarchical and complex designs, identification of the appropriate level for tests and full reporting of outcomes
☒	☐	Estimates of effect sizes (e.g. Cohen's *d*, Pearson's *r*), indicating how they were calculated

Our web collection on statistics for biologists contains articles on many of the points above.

Software and code

Policy information about availability of computer code

Data collection	Whole genome sequencing data were generated using Illumina HiSeq sequencing platform. Isolate metadata was collected and recorded by Scottish Haemophilus, Legionella, Meningococcus & Pneumococcus Reference Laboratory (SHLMPRL), Scottish Microbiology Reference Laboratories (SMiRL). Flow cytometry data was collected using FACSDiva software (v9, BD biosciences). Microscopy images were obtained fusing the Zeiss LSM 710 inverted confocal, recorded using Zen Black 2011 SP7 (V14.0.7.201) software and analyzed using GIMP v2.8.
Data analysis	Reads were trimmed with Trimmomatic (v0.36) using default settings and de novo assembly was performed using SPAdes (v3.10.0) and contigs were reordered with Mauve Contig Reorderer (v2.4.0). Gene annotation and functional prediction for each assembly was generated using Prokka (v1.12). The quality of whole genome sequencing was assessed using Quast (v4.5). Functional prediction of non-synonymous mutations in lag-1 were predicted using PROVEAN (v1.1.3).<br>Genes were translated and aligned at the codon level using TranslatorX and Mafft (v7.271). Phylogenetic networks were generated using uncorrected P-distances with the equal angle method in Splitstree v4.14.6 67.<br>Sequence alignments were visualised in Artemis Comparison Tool v.17.0.1 and figures generated with Easyfig v2.2.2<br>A ML phylogenetic tree was then constructed using RAxML (v8.2.10) from the 1.8 Mbp core genome alignment generated using Parsnp v1.2 (https://github.com/marbl/parsnp)<br>A customised version of the phylogeny-based dataset reduction tool called Treemmer (https://git.scicore.unibas.ch/TBRU/Treemmer)<br>GWAS analysis based on k-mer enrichment was performed using SEER (v1.1.3alpha). Pan-GWAS was performed using the homolog clusters generated by ROARY (v3.12.0) using the following settings (-i 95 -s) 31. The association analyses were calculated using SCOARY (v1.6.9).<br>Flow cytometry data was collected using FACSDiva software (v9, BD biosciences) and the data was analyzed using FlowJo software (v10)<br>GraphPad prism v7 was used to create the figures and to do the statistical analysis of CFU, C3 deposition and flow cytometry data.<br>Figure panels were made using Inkscape v0.92.3.

For manuscripts utilizing custom algorithms or software that are central to the research but not yet described in published literature, software must be made available to editors and reviewers. We strongly encourage code deposition in a community repository (e.g. GitHub). See the Nature Research guidelines for submitting code & software for further information.

Data

Policy information about availability of data

All manuscripts must include a data availability statement. This statement should provide the following information, where applicable:

- Accession codes, unique identifiers, or web links for publicly available datasets
- A list of figures that have associated raw data
- A description of any restrictions on data availability

The sequence data that support the findings of the study have been deposited in the European Nucleotide Archive (ENA) database under Bioproject number PRJEB31628. Individual isolate accession numbers are provided in Supplementary Data 1. A source data file is provided with this paper.

Field-specific reporting

Please select the one below that is the best fit for your research. If you are not sure, read the appropriate sections before making your selection.

☐ Life sciences ☐ Behavioural & social sciences ☒ Ecological, evolutionary & environmental sciences

For a reference copy of the document with all sections, see nature.com/documents/nr-reporting-summary-flat.pdf

Ecological, evolutionary & environmental sciences study design

All studies must disclose on these points even when the disclosure is negative.

Study description	Population genomic study comparing L. pneumophila human clinical and environmental samples.
Research sample	Comparison of 900 L. pneumophila isolates from human clinical and environmental samples. Those include n=400 whole genome sequenced isolates from the SHLMPRL archived collection and n=500 assembled genomes that were available in the NCBI Genbank. Strains from SHLMPRL archive that did not express or express different variants of the lag-1 gene were used in functional studies to test for complement-resistance. Genetically modified strains for lag-1 gene were used to confirmed the observed phenotype in vitro and in a murine model of L. pneumophila pneumonia.
Sampling strategy	No sample size calculation was carried out prior to data collection. We used the genomes of all sequenced isolates from SHLMPRL archive collection until 2015. All experiments to test for L. pneumophila phenotypes in vitro and in vivo were tested with at least 3 biological replicates (plasma, serum, neutrophil donors and mice)
Data collection	Whole genome sequencing data were collected using Illumina HiSeq sequencing platform. Isolate metadata was collected and recorded by Scottish Haemophilus, Legionella, Meningococcus & Pneumococcus Reference Laboratory (SHLMPRL), Scottish Microbiology Reference Laboratories (SMiRL). Flow cytometry data was collected using FACSDiva software v9 (BD biosciences). CFU data was collected in paper and pen and transfered to excel spreadsheets for analysis. C3 deposition data collected using Clariostar Mars data analysis software. Microscopy images were obtained fusing the Zeiss LSM 710 inverted confocal, recorded using Zen Black 2011 SP7 (V14.0.7.201) software and analyzed using GIMP v2.8.
Timing and spatial scale	We used the SHLMPRL archived collection with L. pneumophila samples from 1984 to 2015.
Data exclusions	Isolates belonging to the minor subspecies (L. pneumophila subsp. fraseri, L. pneumophila subspp. pascullei and L. pneumophila subsp. raphaeli) were excluded from the analysis.
Reproducibility	Multiple iterations of the GWAS analyses were performed on random subsets of the genome sequences. Two different approaches (k-mer and gene presence absence) was also in the GWAS study to identify the significant association of genotype with clinical origin. In vitro studies were performed with technical replicates and each experiment was reproduced at least 3 times using independent biological replicates. We observed some variability between donors regarding log CFU values of L. pneumophila in plasma/serum as well as in the levels of bacterial internalization by neutrophils, although the differences between the groups/strains were consistent in the independent biological replicates.
Randomization	The isolates were designated as clinical or environmental based on isolation source as described in the accompanying metadata. To control for the effect of population structure (inherited genotypes), isolates were selected from a diverse range of clinical sources (multiple patients, and locations) and environmental location and subsampled to maximise population diversity.
Blinding	Isolates were collected based on availability in the reference strain collection and public sequence database. The genotype (genome sequence) of each isolate was not known before the association analysis was performed.

Did the study involve field work? ☐ Yes ☒ No

Reporting for specific materials, systems and methods

We require information from authors about some types of materials, experimental systems and methods used in many studies. Here, indicate whether each material, system or method listed is relevant to your study. If you are not sure if a list item applies to your research, read the appropriate section before selecting a response.

Materials & experimental systems

n/a	Involved in the study	
☐	☒	Antibodies
☒	☐	Eukaryotic cell lines
☒	☐	Palaeontology and archaeology
☐	☒	Animals and other organisms
☐	☒	Human research participants
☒	☐	Clinical data
☒	☐	Dual use research of concern

Methods

n/a	Involved in the study	
☒	☐	ChIP-seq
☐	☒	Flow cytometry
☒	☐	MRI-based neuroimaging

Antibodies

Antibodies used

mAb 3/1 from Dresden panel. Polyclonal AlexaFluor 488 conjugated donkey anti-mouse IgG (H+L) (ThermoFisher Scientific, Cat no. A21202). FITC conjugated goat F(ab')2 anti-human C3 complement (Protos Immunoresearch, Cat no. 365)

Validation

mAb 3/1 from the Dresden panel has been frequently used to subtype L. pneumophila strains by the Scottish Microbiology Reference Laboratory. The specificity of the antibody was confirmed by testing known strain that do and do not react with this antibody (controls in Fig. 5a, 5b and Supplementary Fig 6a). Detection of C3 on bacteria surface after serum incubation with FITC conjugated goat F(ab')2 anti-human C3 complement (Protos Immunoresearch) as been previously demonstrated (DOI: 10.7371/ jounal.ppat.1003816).

Animals and other organisms

Policy information about studies involving animals; ARRIVE guidelines recommended for reporting animal research

Laboratory animals

All mice used were 12 - 14 weeks old, female WT on a C57BL/6J background. Mice were either purchased from Charles River or from institutional breeding stock at the Charité - University Medicine, Berlin. Upon transfer to the animal unit, mice were kept in ventilated cages at a conditioned room temperature at 22°C +/- 2°C and humidity at 55% +/- 5%. A dark/light cycle of 12h/12h was maintained. All experiments were approved by the LaGeSo (landesamt für Gesundheit und Soziales) Berlin.

Wild animals

No wild animals were used in the study.

Field-collected samples

No field-collected samples were used in the study.

Ethics oversight

All animal experiments were approved by governmental animal welfare committees by the LaGeSo (Landesamt für Gesundheit und Soziales) Berlin (LAGeSo Berlin; approval IDs G0334/17). All experiments are assigned to project G0334/17.

Note that full information on the approval of the study protocol must also be provided in the manuscript.

Human research participants

Policy information about studies involving human research participants

Population characteristics

Blood was collected from anonymous donors

Recruitment

Recruitment advertisements were be placed on noticeboards throughout the Roslin Institute and the text was circulated by email to the Roslin Institute and Vet Staff email lists. Written informed consent was received from all volunteers participating in the study. No compensation was provided to the volunteers. Sex and age of the donors is unknown. A randomly-generated alphanumeric code was allocated to the sample and no other information was made available to the researchers.

Ethics oversight

Ethical approval for the collection of blood from anonymous donors was granted by the University of Edinburgh Research Ethics Committee. This study was reviewed by the University of Edinburgh, College of Medicine Ethics Committee (2009/01) and subsequently renewed by the Lothian Research Ethics Committee (11/AL/0168).

Note that full information on the approval of the study protocol must also be provided in the manuscript.

Flow Cytometry

Plots

Confirm that:

☒ The axis labels state the marker and fluorochrome used (e.g. CD4-FITC).

☒ The axis scales are clearly visible. Include numbers along axes only for bottom left plot of group (a 'group' is an analysis of identical markers).

☒ All plots are contour plots with outliers or pseudocolor plots.

☒ A numerical value for number of cells or percentage (with statistics) is provided.

Methodology

Sample preparation	Human neutrophils were obtained by Ficoll/Histopaque centrifugation from venous blood from healthy volunteers at the Roslin Institute. The neutrophils were infected with bacteria labeled with FITC or expressing DsRed in the presence or absence of human autologous serum. Infected neutrophils and bacteria alone were fixed in formalin before flow cytometry analysis.
Instrument	BD LSRFortessa X-20 flow cytometer (BD biosciences)
Software	Data was collected using FACSDiva software (v9, BD biosciences) and the data was analyzed using FlowJo software (v10)
Cell population abundance	MFI was evaluated in more than 75% of all acquired events (more than 95% of gated neutrophils).
Gating strategy	Neutrophils were gated using the known FSC/SSC profile and doublets were excluded using FSC-H/FSC-A ratio. MFI of FITC and DsRed was measured in the singlet population. For flow cytometry of bacterial populations, the FSC/SSC gating was defined using fluorescent strains. MFI of FITC and DsRed was measured in the entire bacterial population.

☒ Tick this box to confirm that a figure exemplifying the gating strategy is provided in the Supplementary Information.
